# Efficacy and safety of modified Bushen Yiqi formulas (MBYF) as an add-on to formoterol and budesonide in the management of COPD: study protocol for a multicentre, double-blind, placebo-controlled, parallel-group, randomized clinical trial: FB-MBYF Trial

**DOI:** 10.1186/s13063-022-06057-7

**Published:** 2022-02-14

**Authors:** Qing Kong, Yuxue Cao, Zhen Gao, Jing Sun, Hongying Zhang, Yijie Du, Yubao Lv, Sihan Zhou, Zihui Tang, Baojun Liu, Jingcheng Dong

**Affiliations:** 1 Department of Integrative Medicine, Huashan Hospital, Fudan University, No. 12 Urumqi MIDDLE Road, Shanghai, 200040 China; 2 Institutes of Integrative Medicine, Fudan University, Shanghai, China; 3 Qingpu Traditional Chinese Medicine Hospital, Shanghai, China

**Keywords:** Randomised clinical trial, Design and method, COPD, Traditional Chinese medicine formula, Formoterol and budesonide

## Abstract

**Background:**

Inhaled glucocorticoid corticosteroid (ICS), long-acting β2-adrenoceptor agonist (LABA), and other drugs have limited therapeutic effects on COPD with significant individual differences. Traditional Chinese medicine (TCM)-modified Bushen Yiqi formula (MBYF) demonstrates advantages in COPD management in China. This study aims to evaluate the efficacy and safety of MBYF as an add-on to budesonide/formoterol in COPD patients and confirm the related genes affecting the therapeutic effect in the treatment of COPD.

**Methods:**

In this multicentre, randomised, double-blind, placebo-controlled, parallel-group study, eligible patients with COPD will randomly receive a 360-day placebo or MBYF as an adjuvant to budesonide/formoterol in a 1:1 ratio and be followed up with every 2 months. The primary outcomes will be the frequency, times, and severity of acute exacerbation of COPD (AECOPD), COPD assessment test (CAT) score, and pulmonary function tests (PFTs). The secondary outcomes will include the modified Medical Research Council (mMRC) dyspnoea scale, 6-min walking test (6MWT), BODE index, quantitative scores of syndromes classified in TCM, inflammation indices, and hypothalamic-pituitary-adrenaline (HPA) axis function. We will also test the genotype to determine the relationship between drugs and efficacy. All the data will be recorded in case report forms (CRFs) and analysed by SPSS V.20.0.

**Discussion:**

A randomized clinical trial design to evaluate the efficacy and safety of MBYF in COPD is described. The results will provide evidence for the combination therapy of modern medicine and TCM medicine, and individual therapy for COPD.Trial registration.

**Trial registration:**

ID: ChiCTR1900026124, Prospective registration.

**Supplementary Information:**

The online version contains supplementary material available at 10.1186/s13063-022-06057-7.

## Introduction

Chronic obstructive pulmonary disease (COPD) will become the third leading cause of death worldwide by 2030, with low- and middle-income countries accounting for nearly 90% of COPD deaths [1]. In China, 13.7% of the population over 40 years of age suffers from COPD, and acute exacerbation COPD (AECOPD) is the leading cause of death, creating a sizeable socioeconomic burden [2–5]. COPD is strongly influenced by cigarette smoking and genetic factors [6] and is characterised by a progressive airflow limitation caused by chronic inflammation of the airway and lung parenchyma [7, 8]. Patients with COPD experience lasting respiratory symptoms and systemic manifestations, such as cardiovascular disease, osteoporosis, muscle weakness, depression, and lung cancer, which lead to an impaired quality of life [9, 10]. The current treatment guidelines advocate inhaled corticosteroids (ICSs) and β2-adrenoceptor agonists (LABAs) or long-acting muscarinic antagonists (LAMAs) from the 2021 GOLD guidelines [11]. However, ICS and LABA or LAMA merely provide symptomatic relief and do not effectively relieve oxidative stress or improve the trend of declining pulmonary function [12–16]. Moreover, drug responses vary significantly among individuals. Long-term ICS can be ineffective or even associated with undesirable side effects, including osteoporosis, peptic ulcer bleeding, and hypertension [17–20]. The treatment of COPD that solely relies on modern medicine has encountered a bottleneck [21].

The use of multipronged approaches, including traditional Chinese medicine (TCM), has dramatically increased in response to COPD and its overlapping comorbidities [22]. Traditional Chinese medicine (TCM) has notable advantages in relieving symptoms, improving patients’ quality of life, enhancing efficacy, and reducing the side effects of modern medicine [23–26]. In TCM theory, COPD is considered to be an ageing-related disease, followed by a group of syndromes classified in TCM by deficiency and stasis, which refers to the weakness of the lung, spleen, and kidney during both the stable period and onset period [27]. Recently, real-world evidence-based studies have revealed the predominance of this TCM syndrome known as “Fei_Shen_Qi_Xu” in Chinese individuals [28]. We have observed that Bushen Yiqi formulae (BYF), an herbal formula targeting TCM syndromes, demonstrates effectiveness in ameliorating symptoms, reducing exacerbations, and improving pulmonary function in treating COPD in the clinic [28]. BYF consists of three herbs, as shown in Table 1. As clinical experience has accumulated, we have discovered that modified BYF (MBYF), which combines BYF with two additional herbs, is more effective than traditional BYF in COPD patients.

**Table 1:** Components and dose of MBYF

Formula name		Chinese name	English name	Origin	Weight (g)*	Weight (%)
MBYF	BYF	Huangqi	Astragali radix	The dried root of Astragalus membranaceus (Fisch.). Bge. Var. Mongholicus (Bge.). Hsiao	1.5	14.65
		Yinyanghuo	Epimedii folium	The dried leaf of Epimedium brevicomu Maxim	0.5	4.87
		Dihuang	Rehmanniae Radix	The dried root of Rehmannia glutinosa Libosch	3	29.30
		Huangqin	Scutelllariae radix	The dried root of Scutellaria baicalensis Georgi	2.24	21.87
		Chishao	Peaoniae radix rubra	The dried root of paeonia lactiflora Pall	3	29.30
		Total			10.24	100%

^*^The weight of every single herb in each bag of MBYF in granule form

*MBYF* Modified Bushen Yiqi Formulas

MBYF consists of five herbs, as shown in Table 1 [29–31]. Furthermore, in vivo and in vitro experiments show that MBYF and its compounds can significantly adjust inflammation and immunology [29, 30, 32–35]. Icariin in Yinyanghuo has anti-inflammatory effects, upregulates the amount of GRα, and promotes its nuclear translocation in inflammatory cell models and the lung tissue of inflammation animal models [36, 37]. Astragaloside in Hungqi reduces the protein expression of the JAK3/STAT3/NF-B pathway in CS-induced COPD mice [38]. Scutellaria baicalensis in Huang Qin exerts lung function protection, proinflammatory and anti-inflammatory cytokine regulation, anti-airway remodelling, and antioxidant roles in a long-term CS-induced COPD model [39, 40]. Paeonol in Chi Shao has anti-inflammatory and antioxidant functions against CS-induced lung inflammation in vivo and in vitro [41, 42].

Therefore, we hypothesise that adding MBYF to budesonide/formoterol makes it superior to budesonide/formoterol alone in COPD patients’ exacerbation reduction, symptom amelioration, and pulmonary function improvement. Therefore, we designed a multicentre, double-blind, randomised clinical trial to evaluate the efficacy and safety of MBYF as an add-on to budesonide/formoterol in patients with COPD to provide evidence for a multipronged approach to health care resulting in the adjudication of integrated traditional Chinese medicine (TCM) and orthodox medicine.

In addition, precision medicine has become increasingly popular in recent years in health care, with medical decisions, methods, and interventions based on individualised information [43–45]. At present, evidence for the precise treatment of asthma has been obtained. For example, the detection of the corticotropin-releasing hormone receptor 1 gene (CRHR1) can predict the short-term therapeutic effect of ICS [46, 47]. The FCER2 gene can predict the long-term treatment of ICS [48, 49]. The ADRB2 gene can predict the efficacy of β-agonists and anticholinergic drugs. SABAs or LABAs should not be used for the Arg/Arg type, while SABAs or LABAs can significantly improve lung function for the Gly/Gly type. In contrast, patients with Arg/Arg-type asthma can be treated with tiotropium bromide powder for inhalation: the response rate is as high as 60% [50]. Glucocorticoid-induced transcript 1 gene (GLCCI1) variants (RS 37972C/T and RS 37973A/G) can affect the effect of glucocorticoid therapy in patients [51–53]. Whether or not genes that influence the efficacy of ICS and salmeterol for asthma also influence the efficacy of COPD had become a pressing question. Thus, this study also detected genes that influence the efficacy of ICS/LABA and other drugs in COPD treatment to provide evidence for clinical precision medicine.

## Method and analysis

### Study design

**Fig. 1:**
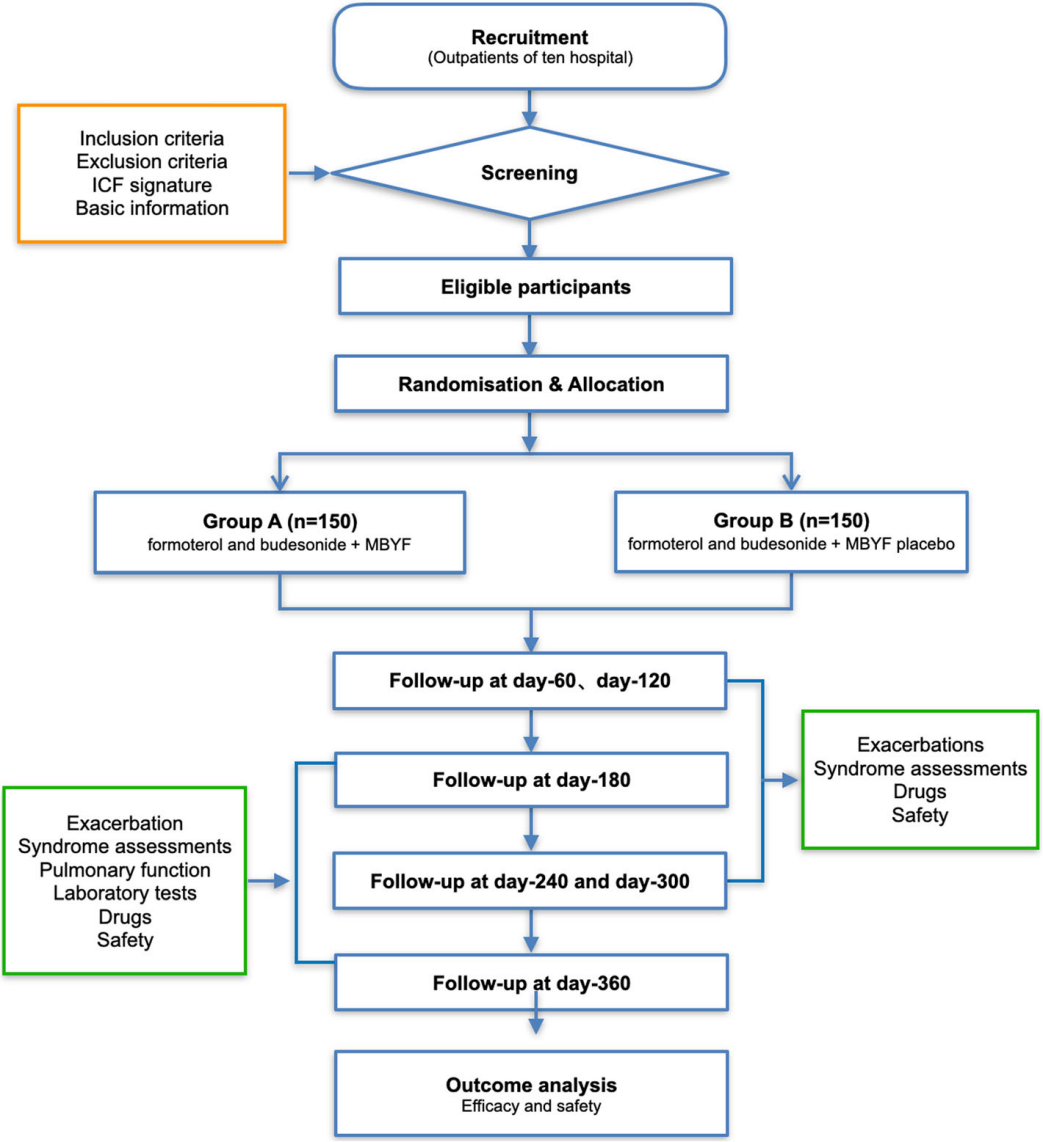
Flowchart of the study

This is a multicentre, randomised, double-blind, placebo-controlled, parallel-group clinical trial. We will rigorously follow the latest Consolidated Standards of Reporting Trials (CONSORT 2017) for Chinese herbal Medicines recommendations [54] and comply with the principles of the Declaration of Helsinki and Good Clinical Practice guidelines. Study protocol is written according to Standard Protocol Items: Recommendations for Interventional Trials and 2013 statement for herbal interventions [55]. A total of 300 patients diagnosed with COPD will be enrolled in the trial, consisting of a 7-day screening period and a 360-day treatment period with every 60-day follow-up. Patients will receive 10.24 g MBYF or placebo in granule form two times per day for 360 days in addition to their routine medication. Figure 1 is the flowchart of the study design.

### Participant recruitment

**Fig. 2:**
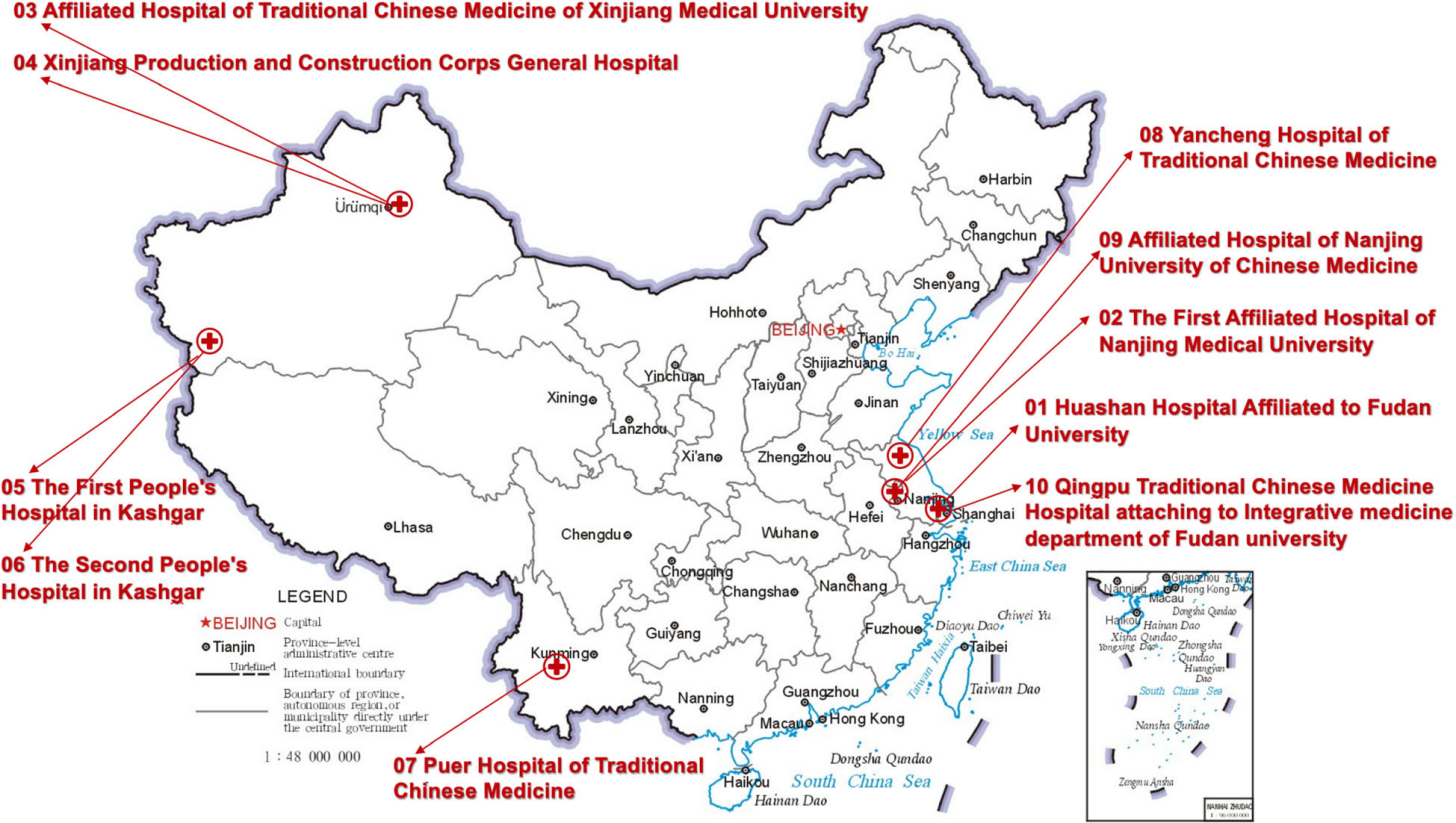
Hospital location

Patients undergoing pulmonary function tests (PFTs) for clinical purposes will be evaluated for entry into the study. Inpatients and outpatients at the ten participating hospitals will be screened according to the inclusion and exclusion criteria by two experienced respiratory physicians separately. Ten hospitals were selected as study centres because they are either attached to universities or have experience in RCTs. As shown in Fig. 2, these hospitals are spread out throughout China, with locations in Shanghai, Yunnan, Xinjiang, and Jiangsu provinces. Clinical physicians and nurses who are qualified in good clinical practice (GCP) and have been trained by the PI will carry out recruitment and follow-up.

The participants will be enrolled in the trial only when confirmed by each of our two respiratory physicians. Additionally, recruitment advertisements for the study will be posted on web pages and notice boards in the ten participating hospitals and resident communities. It includes a brief description of the subjects needed, the medicines, medical examinations, and means of participation in this study. We will record the basic demographic information and reasons for nonparticipation for those who are ineligible or who decline to participate. The first volunteer was recruited on January 14, 2020, in Xinjiang. Due to COVID-19, we expect to complete the recruitment process around December 2021 and report the results as soon as possible.

### Diagnostic criteria of COPD

The diagnosis of COPD will be made according to the 2019 GOLD science committee report in accordance with the comprehensive analysis of clinical manifestations, risk factor exposure history, signs and laboratory examinations, and other data. The main symptoms of COPD are chronic cough, sputum and/or dyspnoea, and risk factor exposure history. A limitation causing incomplete reversible airflow is required for the diagnosis of COPD. The gold standard for COPD is a FEV1/FVC of < 70% after using bronchodilators. Anyone with a history of smoking, exposure to environmental or occupational pollution, and/or cough, sputum, or difficulty breathing will be screened by PFTs. Chest X-ray examination helps determine the extent of hyperinflation in the lungs. Patients with bronchial asthma, bronchiectasis, congestive heart, and other lung diseases, such as heart failure and tuberculosis, were excluded.

### Diagnostic criteria of TCM syndrome

TCM syndrome of “Fei_Shen_Qi_Xu” will refer to “Clinic terminology of traditional Chinese medical diagnosis and treatment-Syndromes, GB/T 16751.2-1997.” Table 2 shows the diagnostic criteria of “Fei_Shen_Qi_Xu.” If patients have two of the primary symptoms and two of the secondary symptoms, then TCM syndrome of “Fei_Shen_Qi_Xu” is determined.

**Table 2:** Diagnostic criteria of TCM syndrome

Primary symptoms	
[1] Shortness of breath	
[2] Cough	
[3] Chest tightness	
Secondary symptoms	
[1] Tinnitus	
[2] Soreness-tired of waist and knee	
[3] Spontaneous perspiration, intolerance to cold, cold hands, and feet	
[4] Pollakiuria	
[5] Lassitude, fatigue, disinclination to talk	

### Inclusion criteria

Inclusion criteria include the following:

1. Patients clinically diagnosed with COPD with airflow limitation included the following: a postbronchodilator fixed ratio FEV1/FVC < 0.7, 30% ≤ FEV1 < 80% predicted;
2. Patients without respiratory tract infection (Patients with nasal congestion, runny nose, sneezing and other upper respiratory tract infections, or who are coughing up yellow sputum, have leukocytes, have an elevated CRP, or who show any pulmonary imaging evidence of acute-stage infection can be excluded) and acute exacerbation of COPD (AECOPD) in the past four weeks;
3. Patients with TCM syndrome of ‘Fei_Shen_Qi_Xu’;
4. Patients with severe exacerbation history: ≥ 2 or **≥** 1 leading to hospital admission in the past year;
5. An age range of 40 to 85 years;
6. Provision of written informed consent by participants or surrogates voluntarily.

### Exclusion criteria

Exclusion criteria include the following:

1. Airflow obstruction caused by asthma and other non-COPD;
2. Patients with lung transplantation or pneumonectomy;
3. Patients needing long-term oxygen therapy (daily oxygen inhalation time ≥ 12 h);
4. Patients with a history of chronic alcoholism or drug abuse;
5. Patients with the standard treatment of oral glucocorticoid at present;
6. Patients with the treatment of pulmonary rehabilitation;
7. Patients accompanied by severe heart diseases, liver and kidney;
8. Patients with a malignant tumour, leukaemia, aplastic anaemia, myelodysplastic syndrome, thrombocytopenia, multiple myeloma and other haematological diseases;
9. Patients who are allergic or intolerant to the study of drugs;
10. Patients who have participated in other clinical trials in the past four weeks; and
11. Pregnant women or breastfeeding women.
12. Other.

### Removal, dropout, and termination criteria

Participants not taking medication during the treatment period will be removed. Participants can voluntarily drop out at any time during the trial. Eligible subjects failing to complete the observation period will be considered dropout cases regardless of the time or reason. Reasons for dropout will be recorded in the case report forms (CRFs), and the last data recorded for these participants will be included in the data analysis. The trial will be suspended if serious adverse events (SAEs) relevant to MBYF occur and if the participant decides to join in another clinical research project regarding COPD, demonstrates hypersensitivity towards MBYF, such as abnormal stomach ache and diarrhoea, suffers from an acute life-threatening disease, or is using a combination of drugs, especially drugs that affect the efficacy and safety of MBYF.

The whole study will be terminated in the following circumstances: [1] masking of the randomisation fails, or [2] the unblinding rate (unblinding cases/300 × 100%) exceeds 20% of the sample size. The assessments of all follow-ups will be completed.

### Randomisation and blinding

A statistician not involved with enrolling participants will generate the random number sequence using SAS. The patients will be randomly divided into the MBYF group or the placebo group in a 1:1 ratio. Blinding will be performed as previously described [31]. Briefly, random numbers will be placed in a closed and opaque envelope without access to directly involved individuals in the study. The PI will deliver “allocating envelopes” that contain the allocating number matched with the drug number to each subcentre. A physician in subcentre will distribute an independent envelope for each participant. To ensure the implementation of the blinding method, participants in the placebo group will be given the same number of placebo drugs and follow the same medication schedule as the treatment group. MBYF and MBYF placebo will be made with the same shapes, smells, and tastes. Trial participants, care providers, outcome assessors, and data analysts will be blinded after the assignment of interventions.

### Procedure for unblinding

After the first administration, if any clinically significant adverse event is potentially related to MBYF treatment, the study physician will reevaluate the participant. The sub-PI or delegate will decide whether an unblinding procedure is necessary. If unblinding is considered necessary, the actual allocation information in the “unblinding envelope” will be provided to the physician. Details including time, place, and reason should be recorded in detail in the CRF.

### Interventions

Budesonide formoterol powder inhaler (160 μg/4.5 μg, once in the morning and evening, 2 inhalations each time) will be provided as a conventional treatment to all participants according to the GOLD for COPD. MBYF or placebo (10.24 g/bag, one bag at a time, two times per day, 360 days) will be provided as an adjunct medication randomly using an envelope. MBYF and placebo (containing 10% MBYF, maltodextrins, taste-masked agents, and colour correction agents) are identical in taste and appearance. The Huarui Sanjiu pharmaceutical industry produces them in Shenzhen, China. Granule production was certified to the standard certification of the National medical Products Administration in China (https://www.nmpa.gov.cn/directory/web/nmpa/xxgk/ggtg/qtggtg/20210210145453181.html). The choice of placebo as a comparator conforms with the purpose of this study, which is mainly to determine the effectiveness and safety of MBYF based on stable-stage COPD routine treatments. Two-month follow-ups are based on our previous clinical studies on COPD interventions.

### Intervention modifications

According to the requests from participants, the PIs in each centre will decide the intervention’s modification or discontinuation when AECOPD occurs, which indicates the need for ICU admission, or when unexpected adverse effects happen.

### Adherence to treatment

Free MBYF will be contributed to subjects every 2 months. All unused packs of drugs and empty bags will be returned to the investigator. Additionally, a convenient WeChat group online (a popular smartphone app for instant messaging) will be built for information exchange between investigators and participants or their family members. Some educational videos and articles for inhaler use will be shared online. Doctors working at outpatient departments will give a health opinion to participants during follow-up, which will be helpful for participants’ adherence. Adherence will be determined by counting drugs or empty bags as follows: Medication adherence% = [actual dose/(specified daily dose days)] 100%. Total medication consistency ranging from 80 to 120% will be eligible for the protocol analysis set. Parts of the laboratory tests will be freely performed on a standard schedule, which aids in monitoring adherence.

### Concomitant care

When comorbidities threaten patients’ lives, any form of treatment will be permitted. The outcome will be measured when patients recover to a stable condition and continue taking drugs for 2 weeks. Other ICSs and bronchodilators are prohibited during a stable period. Herbal drugs not related to “Tonifying Shen” are allowed to be used according to the needs of the disease. If MBYF causes other severe comorbidities, then the PI will afford the portion of the fees for the hospital admission.

### Data collection

Items to be measured and the time window of data collection are shown in Table 3. Demographic information, medical history, concomitant medication, etc., will be collected at baseline. Laboratory test data from the subcentre will be adjusted according to Huashan Hospital standards for analysis. Patients will answer questionnaires without incentives. To promote participant retention and complete follow-up, enrolled participants will join a WeChat group where physicians will provide free consultations. Participants and their families will be informed that standardised treatment is beneficial in reducing COPD exacerbations, lowering medical costs, and maximising benefits. Telephone calls will be conducted every month.

**Table 3:** Flow diagram for MBYF clinical trial

	Enrollment	Allocation	Post-allocation					
**Timepoint**	− 7 days	0 ± 3 days	60 ± 3 days	120 ± 3 days	180 ± 3 days	240 ± 3 days	300 ± 3 days	360 ± 3 days
**Enrolment:**								
Eligibility screen	X							
Informed consent	X							
Background information	X							
**Interventions**								
MBYF or placebo								
Budesonid and Formoteraol								
**Safety outcomes**								
ECG		X			X			X
Chest X-ray or CT		X			X			X
Laboratory examinations*		X			X			X
AEs		X			X			X
**Primary outcomes**								
The frequency、time and severity of AECOPD		X	X	X	X	X	X	X
CAT score		X			X			X
Pulmonary function test		X						X
**Secondary outcomes**								
mMRC dyspnea scale		X			X			X
6MWT		X			X			X
BODE index		X			X			X
Quantitative score of TCM syndrome		X			X			X
Inflammation indexes and HPA axis function		X						X
**Other test**								
Genotypes		X						X

*MBYF* modified Bushen Yiqi formulas, *AECOPD* acute exacerbation of COPD, *CT* computed tomography, *AE* adverse event, *ECG* electrocardiogram, *CAT* COPD assessment test, *mMRC* Modified Medical Research Council, *6MWT* Six minutes of walking test, *BODE* B: body mass index (BMI), D: Degree of airflow obstruction, E: Evaluation of dyspnea, D: Dynamic ability, *TCM* traditional Chinese medicine, *HPA* hypothalamic-pituitary-adrenaline axis

Laboratory examinations*: blood and urine routine, liver and kidney function, glucose

### Data management and monitoring

All the information in the CRF will be saved in a specialised clinical experimental database. The data management procedures are similar to those of the other protocols that we have published (https://www.ncbi.nlm.nih.gov/pubmed/32883322). The data monitoring committee comprises an investigator from Huashan Hospital due to the lack of sponsors. A data monitoring committee will audit the trial conducted via internet meeting monthly and site investigation semiannually. An interim analysis will be conducted to evaluate the safety of MBYF when 30 subjects have been followed up with at 180 days. PIs and the ethics committee will have access to these results and decide when to terminate the trial.

### Background information

Background information included demographic data (sex, age, height, weight, and so on) and general clinical data (medical history, course of the disease, treatment history, combined diseases, concomitant medications, and so on), which will be recorded during the 1-week screening period. The participants’ information and privacy will be strictly protected and not released to the public.

### Safety outcomes

Safety will be assessed at 180 and 360 days after randomisation by electrocardiogram (ECG), chest X-ray or computed tomography (CT), laboratory examinations (routine blood and urine tests, liver and kidney function, glucose), and adverse events (AEs). AEs will be documented and monitored during the treatment until they disappear.

### Primary outcomes

1. The frequency, time, and severity of AECOPD

The frequency, time, and severity of AECOPD will be collected every 60 days after randomisation. AECOPD is defined as a patient presenting with two of the following symptoms (at least one primary symptom) for at least 2 days. Primary symptoms include the following: dyspnoea, profuse sputum, purulent sputum. Secondary symptoms include the following: wheezing, pain in the throat, cough, common cold. The interval of AECOPD between two episodes is defined as at least a week. The time of AECOPD is defined as the duration from acute exacerbation to the apparent improvement of the symptoms (the patient’s self-sensation). The severity of AECOPD is defined as following three degrees: mild symptoms either requiring no need for clinical service or oral intake of hormones, antibiotics and oxygen therapy as necessary; moderate symptoms where clinical service is necessary; and oral intake of hormones, antibiotics, or oxygen therapy is required; and severe symptoms where hospitalisation or emergency treatment is needed.

(2) COPD assessment test (CAT) score

The CAT score will be used to evaluate symptoms at baseline and 180 days and 360 days after randomisation. Please see the supplementary file.

(3) Pulmonary function tests(PFTs)

A postbronchodilator forced expiratory volume in 1 s (FEV1) % predicted the forced vital capacity (FVC) ratio (FEV1/FVC), and blood gas analysis will be measured at baseline 360 days after randomisation. Before the test, treatment drugs should be discontinued for more than 6 h, intense activity should be avoided for 2 h, and patients should rest for 15 min.

### Secondary outcomes

1. The Modified Medical Research Council (mMRC) dyspnoea scale mMRC will be used to evaluate dyspnoea at baseline and 180 days and 360 days after randomisation. Please see the supplementary file.

(2) Six-minute walking test (6MWT)
  The 6MWT will be used to evaluate aerobic exercise capacity at baseline and 180 and 360 days after randomisation. The patient will be asked to walk quickly for 6 min, and the distance will be recorded. Dyspnoea scales, fatigue, respiratory rate, heart rate, and oxygen saturation before and after walking were calculated. The Borg scale will be used to evaluate the severity of dyspnoea and fatigue. Please see the supplementary file.
(3) BODE index
  BODE will be used to predict the mortality rate at baseline and 180 and 360 days after randomisation. It includes the following aspects: [1] B: Body mass index (BMI), [2] D: Degree of airflow obstruction, [3] E: Evaluation of dyspnoea, [4] D: Dynamic ability. The four scores were added together to obtain the BODE score. Please see the supplementary file.
(4) Quantitative scores of TCM syndrome
  Quantitative scores of TCM syndrome will be used to evaluate the TCM syndrome of Fei_Shen_Qi_Xu at baseline and 180 and 360 days after randomisation. The treatment index (*n*) will be calculated as follows: (scores before treatment − scores after treatment)/scores before treatment 100%. Please see the supplementary file.
(5) Inflammation indices and hypothalamic-pituitary-adrenaline (HPA) axis function
  Inflammation indices and HPA axis function will be evaluated at baseline and 360 days after randomisation. Ten millilitres of blood will be drawn, and the serum (2–3 ml) will be separated and sent to Huashan Hospital for TNF-α detection. Five millilitres of whole blood will be drawn, and blood plasma (1–2 ml) will be separated to detect hydrocortisone.

### Other tests

A total of 5 mL of blood will be drawn at baseline, and peripheral blood mononuclear cells will be isolated and stored at − 80 °C in the Huashan Hospital lab of the Integrative Medicine Department to detect CRHR1, FCER2, GLCCI1, and another genotype for future research. The relationship between drug efficacy and genotype will be evaluated.

### Sample size calculation

According to the primary outcomes in the previous trial [24, 28], at the 5% significance level, a total of 118 patients per group will be required for a 2-group, 1-sided calculation to achieve 95% power and the differences of (10.30 ± 6.31, 12.95 ± 5.99) in CAT mean score between the TCM treatment group and placebo group, as calculated using G-power software (latest version. 3.1.9.6, Heinrich-Heine-Universität Düsseldorf, Düsseldorf, Germany). A loss of 15–20% to follow-up is predicted based on experience. This increases the sample size to 142 participants per group. Based on a 1:1 ratio, the final decision is 300 in total.

### Adverse events (AEs)

AEs are defined as negative or unintended clinical manifestations following treatment. Patients will be asked to report to the investigators any abnormal reactions occurring at any time during the trial. In addition, investigators will collect information about abnormal reactions every 60 days. If an AE occurs, the doctor will immediately report it to the PIs and the ethics committee within 24 h. All details include the time of occurrence, degree and duration of AEs, suspected causes, and effective measures and outcomes recorded on CRFs. Subjective discomfort, abnormalities on ECG, chest X-ray or CT, and laboratory tests should be taken seriously. Careful analysis and immediate measures will be taken to protect the safety of the subjects until the AEs disappear.

If AECOPD occurs, patients will be sent to the emergency room and receive standard treatments according to the GOLD guidelines. Aside from AECOPD, the gastrointestinal reaction merits special attention. Patients who suffer from nausea and vomiting due to herbal treatment will have their treatment suspended for 1 week. The investigator will evaluate their ability to continue to participate.

### Quality control of data

CRFs will be used for data collection. All investigators will receive pretrial training on patient screening, data filling, medication use, AE reporting, and other matters. A trial inspector will visit each hospital regularly to check the electronic database and ensure that the protocol is strictly followed. The PIs will be in charge of data validation and take measures to keep the dropout rate below 15%.

### Planned analysis

Baseline data and primary and secondary outcomes will be analysed using the modified intention-to-treat (MITT) population, including those who deviate from the protocol, to guarantee that the groups of patients being compared have similar characteristics. The safety outcomes will be analysed using the safety analysis population (SAP). The Kolmogorov–Smirnov test will be used to determine whether continuous variables follow a normal distribution. The abnormal distribution of variables will be log-transformed to approximately normal distributions before analysis. Continuous variables will be expressed as the mean ± SD or the median, while categorical variables will be shown as counts and percentages. Differences between the two groups will be compared using analysis of variance (ANOVA) for continuous variables and the *χ*^2^ tests for categorical variables. Fisher’s least significant difference test will be used to further explore and compare the mean of the two groups. Analysis of covariance will be used to control for potential confounding variables. A statistical package for social sciences for Windows version 16.0 (SPSS, Chicago, IL, USA) will be used for analysis. Two-sided tests will be used throughout, with the statistical significance level set at 0.05, and 95% CIs will be used for interval estimation. The Bonferroni correction will be used to adjust the *p*-values for multiple primary outcomes [56]. The methods of analysis of each variable are summarised in Table 4. For the primary outcomes, ANCOVA will be used to compare continuous variables including the frequency and days of AECOPD, the score of CAT, the FEV1% predicted, and FEV1/FVC between two groups; *χ*^2^ test will be used to compare categorical variables of the mild, moderate, and severe of AECOPD between two groups. For secondary outcomes, ANCOVA will be used to compare continuous variables including the distance of 6MWT, the score of BODE index and TCM syndromes, the level of TNF-α, IL-6, IL-8, IL-10, ACTH, CRH, CORT, etc.; *χ*^2^ test will be used to compare categorical variables of 0–5 in mMRC dyspnoea scale.

**Table 4:** Variables and method of analysis

Outcomes	Hypothesis	Variables	Analysis method
The frequency and time of AECOPD	MBYF improved outcomes	Frequency and days (continuous)	ANCOVA
The severity of AECOPD		Mild, moderate, severe (categorical)	*χ*^2^ test
CAT score		Score (continuous)	ANCOVA
Pulmonary function		FEV1%predicted, FEV1/FVC, (continuous)	ANCOVA
Blood gas analysis		Pa (CO_2_), Pa(O_2_) (continuous)	ANCOVA
mMRC dyspnea scale		0–5 (categorical)	*χ*^2^ test
6MWT		Distance (continuous)	ANCOVA
BODE index		Score (continuous)	ANCOVA
Quantitative scores of TCM syndrome		Score (continuous)	ANCOVA
Inflammation indexes		TNF-α, IL-6, IL-8, IL-10, etc. (continuous)	ANCOVA
HPA axis function		ACTH, CRH, CORT (continuous)	ANCOVA

### Ethics and dissemination

This study was approved by the Ethics Review Committee of Huashan Hospital, Fudan University, Shanghai, China (Provisional Trial No. (425) 2018) and registered at the Chinese Clinical Trial Registry at http://www.chictr.org.cn/ 15 December 2019 (ChiCTR1900026124). Ethics Review Committee will conduct an independent review of the research to maintain participant safety and welfare. Clinical doctors will obtain informed consent from patient volunteers, usually in outpatient settings. Written informed consent, including additional consent provisions for collecting and using participant data and biological specimens in further ancillary studies, will be acquired from all patients before treatment. Protocol modifications will be conveyed to all subcentres via regular online and offline meetings and phone conversations. All staff involved in the trial will be notified, trained, and qualified again through ZOOM meetings. Case report forms, laboratory specimens, evaluation forms, reports, etc., will be stored in locked file cabinets. All records will be secured with a password-protected access system. The participants’ personal information will be deleted from all study documents and will not be released outside the study without the participants’ approval. The principal investigator (PI) will access the redacted datasets. Site investigators will have direct access to their own site’s datasets and access other sites’ data by request. Participant-level datasets will be available to outside investigators at the end of the trial by request for sharing purposes. The investigators, who are also the authors and who are all doctors in the clinic, will be responsible for initiating, managing, and applying for funding for clinical trials. Substantive contributions to conducting, interpreting, and reporting a clinical trial will be recognised by granting authorship on the final trial report. There is no intended use of professional writers.

A series of measures will be conducted before and after the treatment, including subjective descriptions and laboratory tests focusing mainly on liver and kidney damage, to assess the safety of MBYF from enrolment through the follow-up period. In addition, we will ask every patient by WeChat or phone call about their condition when they finish the trial 2 months later. If adverse events caused by study drugs occur, some relevant examinations and appropriate treatments will be provided for free immediately.

## Discussion

In this multicentre, randomised, double-blind, placebo-controlled clinical trial, we will clarify a practical method to identify the efficacy and safety of MBYF as an adjunct to budesonide/formoterol in the treatment of COPD. A simple, convenient, inexpensive, and effective herbal formula based on TCM syndrome differences might strongly support and enrich COPD management. The level of the HPA axis and pro-/anti-inflammation between the MBYF and placebo groups might indicate the mechanism of TCM treatments on COPD or other ageing diseases. Exploring the relationship between genotype and ICS/LABA/other drugs will provide robust evidence and guidelines for COPD individual therapy. Overall, this finding can form a unique diagnosis and treatment scheme which combines traditional Chinese medicine and modern medicine and promotes its application.

However, there are some limitations to this protocol. First, we choose one of the specific TCM syndromes as the criteria, thus the result is hard to extend to the entire patient with COPD exacerbation. Second, measurement bias and the subjectivity of the questionnaire assessment are inevitable, which may be exacerbated by the loss of some participants during follow-up. Third, the trial will be conducted in ten hospitals in China, and whether similar effects are available to other ethnic groups and regions will remain uncertain. Fourth, although all patients are required to show the negative result of the COVID-19 nuclear test before enrolment, new measures are lacking to better address the unprecedented challenge of carrying out RCTs.

## Trial status

Our trial enrolled 150 volunteers from January 14, 2020, to the present. We have modified the protocol according to the practice and standard protocol items [55, 57]. The new protocol was reported to all subcentres of PIs in a group meeting. Due to COVID-19, we expect to complete the recruitment process around November 2021 and report the results as soon as possible.

## Supplementary Information

**Additional file 1.** Questionnaire Scales of CAT, mMRC, BODE, TCM syndrome.
