# Supplementary material for: Efficacy and safety of modified Bushen Yiqi formulas (MBYF) as an add-on to formoterol and budesonide in the management of COPD: study protocol for a multicentre, double-blind, placebo-controlled, parallel-group, randomized clinical trial: FB-MBYF Trial
Source: Trials. 2022 Feb 14;23:143. doi: 10.1186/s13063-022-06057-7 (PMC8842909; doi:10.1186/s13063-022-06057-7)
Supplement: Supplementary file 1 — Additional file 1. Questionnaire Scales of CAT, mMRC, BODE, TCM syndrome. [file 13063_2022_6057_MOESM1_ESM.pdf]

CAT™ ASSESSMENT

For each item below, place a mark (x) in the box that best describes you currently.
Be sure to only select one response for each question.

			SCORE
**EXAMPLE: I am very happy**	(0) (X) (2) (3) (4) (5)	**I am very sad**	
I never cough	(0) (1) (2) (3) (4) (5)	I cough all the time	
I have no phlegm (mucus) in my chest at all	(0) (1) (2) (3) (4) (5)	My chest is completely full of phlegm (mucus)	
My chest does not feel tight at all	(0) (1) (2) (3) (4) (5)	My chest feels very tight	
When I walk up a hill or one flight of stairs I am not breathless	(0) (1) (2) (3) (4) (5)	When I walk up a hill or one flight of stairs I am very breathless	
I am not limited doing any activities at home	(0) (1) (2) (3) (4) (5)	I am very limited doing activities at home	
I am confident leaving my home despite my lung condition	(0) (1) (2) (3) (4) (5)	I am not at all confident leaving my home because of my lung condition	
I sleep soundly	(0) (1) (2) (3) (4) (5)	I don't sleep soundly because of my lung condition	
I have lots of energy	(0) (1) (2) (3) (4) (5)	I have no energy at all	

TOTAL SCORE:

Reference: Jones et al. ERJ 2009; 34 (3); 648-54.

FIGURE 2.3

MODIFIED MRC DYSPNEA SCALE[a]

PLEASE TICK IN THE BOX THAT APPLIES TO YOU | ONE BOX ONLY | Grades 0 - 4

**mMRC Grade 0.**	I only get breathless with strenuous exercise.	☐
**mMRC Grade 1.**	I get short of breath when hurrying on the level or walking up a slight hill.	☐
**mMRC Grade 2.**	I walk slower than people of the same age on the level because of breathlessness, or I have to stop for breath when walking on my own pace on the level.	☐
**mMRC Grade 3.**	I stop for breath after walking about 100 meters or after a few minutes on the level.	☐
**mMRC Grade 4.**	I am too breathless to leave the house or I am breathless when dressing or undressing.	☐

[a] Fletcher CM. BMJ 1960; 2: 1662.

TABLE 2.5

1-10 Borg Rating Perceived Excertion Scale	
Rest	0
Really Easy	1
Easy	2
Moderate	3
Soft of Hard	4
Hard	5
	6
Really Hard	7
	8
Really,Really,hard	9
Maximal: Just like my hardest race	10

Index	BODE index score			
	0	1	2	3
FEV1 （% of predicted）	≥65	50-64	36-49	≤35
6mWD （m）	≥350	250-349	150-249	≤149
mMRC （scale）	0-1	2	3	4
BMI （kg/m2）	＞21	≤21v		

Quantitative scores of TCM syndrome

Symptoms		Normal	Mild	Moderate	Severe
	Score	0	2	4	6
Main symptoms	Shortness of breath	No	Mild short breath without affection on normal life and work	Short breath with affection on normal life and work	Short breath even when the patient is at ease with significant affection on normal life and work
	Cough	No	Relatively mild	Occasionally	Frequently
	Chest thightness	No	Relatively mild	Occasionally	Frequently
	Score	0	1	2	3
Secondary symptoms	Tinnitus	No	Occasional tinnitus	Frequent tinnitus	Refractory, unstopped tinnitus
	Soreness-tired of waist and knee	No	Relatively mild	Occasionally	Frequently
	Spontaneous perspiration, intolerance to cold, cold hands and feet	No	Slightly moist skin, fear of cold with cold limbs	Moist skin,severe fear of cold with cold body which cannot be relieved by warmth	Perspiration, mild fear of cold
	Pollakiuria	No	Twice night urination	Three times of night urination	More than four times of night urination
	Lassitude, fatigue, disinclination to talk	No	Seldom speak	Unwilling to speak	Reluctant to speak
Total scores					
